# When primary healthcare meets queerstory: community-based system dynamics influencing regional/rural LGBTQ + people’s access to quality primary healthcare in Australia

**DOI:** 10.1186/s12889-023-15289-4

**Published:** 2023-02-23

**Authors:** James J. Lucas, Rojan Afrouz, Andrew D. Brown, Sarah Epstein, Joleen Ryan, Joshua Hayward, Sharon L. Brennan-Olsen

**Affiliations:** 1 School of Health and Social Development, Deakin University, Geelong, VIC Australia; 2 Social Determinants of Health Domain, Institute for Health Transformation, Deakin University, Burwood, VIC Australia; 3 Global Obesity Centre (GLOBE), Institute for Health Transformation, Deakin University, Geelong, VIC Australia; 4 National Indigenous Knowledges, Education, Research, Innovation Institute, Deakin University, Waurn Ponds, Victoria, Australia

**Keywords:** Queer, LGBTQ +, Primary healthcare, Systems thinking, Community, Equity, Regional/rural

## Abstract

**Background:**

Lesbian, gay, bisexual, transgender, Queer, and people of any other minority sexuality or gender identity (LGBTQ + or “Queer”) are often marginalised from accessing quality primary healthcare (PHC) in their local community. This is largely due to Queerphobic, cis-heteronormative/sexist systems pathologising Queer life and identities. The study aims were to: (1) identify key priorities for increasing Queer people’s access to quality PHC as told by Queer people themselves, (2) identify the feedback loops that reduce or support Queer people’s access to quality PHC in non-metropolitan, regional/rural communities, and (3) identify potential action areas to improve system structures to increase Queer people’s access to quality PHC.

**Methods:**

Group Model Building (GMB) workshops were held with a small group (*n* = 8) of LGBTQ + people in regional Victoria with lived experience of using PHC services. This participatory approach permits exploration and visual mapping of local structures causing behaviour patterns of community concern over time – in this case, Queer people’s ability to access quality PHC in the Geelong-Barwon region. This is the first study that specially applies GMB in Queer PHC in the non-metropolitan regional/rural context.

**Results:**

Key community identified PHC priorities were: (a) providers’ level of Queer Literacy, (b) the responsibility of Queer Advocacy (at individual, systemic, and collective levels), (c) support from safe Queer Spaces, (d) strength from a Queer Presence, and (e) power from Intersectional Queer Life. These priorities interconnected, creating system-level feedback loops reinforcing barriers and enablers to Queer people’s access to quality PHC in the Geelong-Barwon region; with potential action areas identified.

**Conclusions:**

Improving Queer people’s access to quality PHC in the Geelong-Barwon region requires embedding principles of Queer Literacy, Queer Advocacy, Queer Space, Queer Presence, and Intersectional Queer Life within practices and service systems. The study findings were distilled into a novel, preliminary set of Queer Equity Principles. These need to be taken back to regional Queer communities for further co-design and planning for translation across PHC practices and systems, with potential applicability in other areas of the healthcare spectrum.

## Background

Primary healthcare (PHC) is an important “first-point-of-call” for people to access community-based health and wellbeing services, while also serving as a referral broker for secondary and tertiary public healthcare systems [1]. The The World Health Organization [2] described PHC as an approach to provide physical, mental, and social health care in the community. In Australia, PHC predominantly takes the form of General Practice Clinics, Community Health Centres, and Allied Health Centres; with the General Practitioner (GP) being the main broker of referrals to secondary and tertiary health services. No referral is needed to access the GP whose focus is the prevention of physical and mental ill-health, alongside reducing the likelihood of hospitalisation [3]. Access to quality PHC is an important public health approach to supporting LGBTQ + people’s health and wellbeing.

Access to quality PHC however, remains a challenge for lesbian, gay, bisexual, transgender, Queer, and people of any other minority sexuality or gender identity (e.g., two-spirit or third gender) (LGBTQ + or “Queer”^1^) in Australia. In Australia, approximately 83% of LGBTQ + people during 2019 accessed a mainstream medical clinic for PHC services, with 25% within a mainstream clinic known to be LGBTQ + inclusive. Only around 6% of LGBTQ + people in Australia accessed a clinic for PHC that caters only for LGBTQ + people [4]. The lack of LGBTQ + -run or friendly PHC places LGBTQ + at risk of facing discrimination and oppression when seeking support from mainstream PHC services due to its history of cis-heteronormative or cis-heterosexist foundations (see [5, 6]). Rosati, Pistella [7] described cis-heteronormativity as “the assumption that all people are heterosexual and their gender identity matches with their birth-assigned sex (cis-gender)” (p. 2). Cis-heteronormativity is interrelated with the term: cis-heterosexism, which refers to “indicating a shared beliefs system according to which heterosexual/cisgender people are considered more natural, real, and authentic than non-heterosexual/trans people” (Rosati et al., 2020, p. 2). The existing literature suggests that PHC’s cis-heteronormative/cis-heterosexist foundation renders invisible LGBTQ + people’s unique health and wellbeing needs, as well as their intersectional identities and lived experiences [8–10] – that is their “Queerstory”. The outcomes of which can include reduced help seeking and delaying care, a lack of health promotion and care that is LGBTQ + inclusive, and fear of disclosing one’s sexuality or gender identity [4, 11–14]. PHC’s cis-heteronormative/sexist foundation also creates challenges for LGBTQ + health workers who, along with their service users, struggle with oppressive power dynamics that make disclosure of their identities and lived experiences (i.e., their Queerstory) unsafe and a resultant fear of using PHC themselves [6]. As a consequence, promotion of Queer-friendly and Queer-run PHC services is inhibited with solutions to such barriers often lying beyond the capacity of individual LGBTQ + health workers and services users [9]. Thus, LGBTQ + people face a range of inequities in accessing quality PHC that also contribute to an associated loss of belonging and social connectedness in their local communities and within PHC systems [10].

Efforts to redress the effects of cis-heteronormativity/sexism in PHC systems on LGBTQ + peoples include development of LGBTQ + inclusion guidelines and best-practice recommendations for PHC services (e.g., Australia’s Rainbow Tick Accreditation Program; see: https://rainbowhealthaustralia.org.au/rainbow-tick). Endorsed by Governments and professional-bodies (e.g., Royal Australian College of General Practitioners), these specific and multifaceted guidelines and best-practice recommendations have begun to create a more inclusive healthcare system for LGBTQ + people [15]. There are limitations with such efforts, in particular the Rainbow Tick Accreditation, in that they do not result in system-level changes nor do much to change day-to-day PHC practices with the Rainbow Tick becoming more a “buzz word” rather than reflecting genuine LGBTQ + inclusivity [16]. As a result, further work is needed to support inclusion of LGBTQ + communities in Australia.

In Australian non-metropolitan regional/rural areas, as classified under the commonly utilised Australian Modified Monash Model of remoteness classification (see: https://www.health.gov.au/sites/default/files/documents/2020/07/modified-monash-model-fact-sheet.pdf), people face barriers to accessing quality PHC including an inadequate number of health care providers in their local community, geographical distance, and general social isolation [6]. The presence of Queer-friendly and Queer-run PHC services are in even less supply. As such, LGBTQ + in regional/rural areas seeking quality PHC face an additional layer of barriers at the geographical level. There are further barriers, at least in the Australian context, whereby regional communities generally tend to hold more conservative values and cis-heteronormative assumptions of sexuality, gender identity, and family structures compared with their metropolitan counterparts [17, 18].

Given that all people’s health is “shaped by access to power and resources based on sex, gender identity, and sexual orientation, as well as other intersecting social categories” [19], further work is also needed in expanding and reshaping the LGBTQ + inclusion narrative in PHC towards one of Queer Equity [20] – that is, ensuring all LGBTQ + people have the power and resources necessary to "attain their full potential for health and wellbeing” [21]. While LGBTQ + inclusion is a necessary start to achieving equity for LGBTQ + people accessing quality PHC, Queer Equity is a next-step evolution as it permits identification and redressing of system-level drivers of inequality in LGBTQ + health and wellbeing outcomes. As with global campaigns focusing on the more inclusive “marriage equality” rather than “same-sex marriage” [22], the focus of debate requires moving away from LGBTQ + people seeking inclusion within cis-heteronormative/sexist PHC service systems and instead demand PHC providers redress the dynamics created by local systems marginalising LGBTQ + people’s access to their service. These changes too are held accountable to LGBTQ + people’s Queerstory, that is their lived experiences and intersectional identities, in regional/rural areas. In this way, an equitable PHC for LGBTQ + involves “the absence of unfair, avoidable or remediable differences among groups of people, whether those groups are defined socially, economically, demographically, or geographically or by other dimensions of inequality (e.g. sex, gender, ethnicity, disability, or sexual orientation)” [21].

The way that cis-heteronormativity/sexism in PHC, the marginalisation of LGBTQ + people, and accountability to LGBTQ + people’s Queerstory reinforce one another is an example of a system-level feedback loop, one type of driver of complexity. System dynamics, one method of systems thinking, identifies feedback loops such as these to understand the drivers of complex problems and identify possible solutions [23]. A first step, therefore, is working with LGBTQ + people to identify priority issues in accessing quality PHC services, identify the underlying feedback loops driving the issues, and identify possible solutions or actions to build toward Queer Equity.

### Aim

The aim of this study was to:

1. identify priority issues influencing regional LGBTQ + people’s access to quality PHC (e.g., GP Clinics and Community Health Centres) as told by LGBTIQ + people themselves
2. identify the regional community-based, system-level feedback loops that reduce or support LGBTQ + people’s access to quality PHC, and
3. identify potential action areas to improve system structures to increase regional LGBTQ + people’s access quality PHC.

### Research team positioning

The Queer-led research team is a diverse group of LGBTQ + identifying people and their allies across various intersections of gender, sexuality, culture, and academic discipline; all of whom hold a genuine commitment to the promotion of LGBTQ + health and wellbeing equities and challenging oppressive cis-heteronormative social norms. The research team worked reflexively in reviewing the process and outcomes from the study to reduce unconsciously imposing our own biases and assumptions; and to hold each member of the research accountable to “doing justice” to the LGBTQ + voices in the study. Community-based system dynamics, a subset of system dynamics, is particularly well suited to partner with communities in addressing complex problems [24]. The research team were therefore committed to remaining mindful of the benefits and challenges of conducting research from an LGBTQ + /ally insider–outsider perspective [25]. In this way, this project represented a Queer-led, community-based initiative focused on promoting equity outcomes for LGBTQ + people in regional/rural communities.

## Methods

### Design

A Community-Based System Dynamics (CBSD) approach [24] was applied through two Group Model Building (GMB) workshops [26]. This participatory research approach permits exploration and visual mapping of the local structures that cause a pattern of behaviour of community concern [26]. GMB has always emphasised the importance of the key stakeholders within a system, but CBSD takes this approach further by increasing community participation in the modelling process through shared facilitation [24].

Several studies have showcased CBSD’s ability to effectively engage people living in rural and regional areas, with at least one study has applied CBSD specifically to rural healthcare access [27–32]. CBSD’s promise as a method to explore social justice issues, such as structural racism [33, 34]. This previous work in CBSD shows its potential to engage people living in rural or regional areas, consider barriers and enablers to healthcare access, and consider structural drivers of oppression and health inequity; therefore, demonstrating the potential of applying CBSD to the current study. CBSD is meant to be a long-term process of capacity building with community [24], and so its application in the current study was considered an initial exploration of the methods with this particular population.

In the current study, the primary area of concern was how LGBTQ + people’s access to quality PHC has changed over time in regional/rural communities of Victoria’s Geelong-Barwon Region (see: https://www.rdv.vic.gov.au/victorias-regions/barwon-south-west). For example, access may have decreased, or increased but slower than expected, or not at all increased despite community efforts. The use of CBSD as a framework for the implementation of GMB as a community-oriented participatory research method has been successful in previous studies (e.g., [30, 32, 35, 36]).

### Participants

A convenience sample of seven LGBTQ + community members participated in the study. All participants were 18 years of age or older and lived in the Geelong-Barwon Region of the Australian state of Victoria. While efforts were made to diversify the sample as much as possible, such as promoting the study via the research team’s local LGBTQ + networks and organisations, participants were also predominantly cis-female, lesbian, White, and of professional class. One participant’s identity included genderqueer, two included bisexual. No further demographic information was collected to protect the confidentiality of participants’ identity within the local Geelong-Barwon communities within which the participants live.

### Ethics and recruitment

Ethical approval for the conduct of the study was granted by the University’s Human Research Ethics Committee. Participants were recruited via the research team’s networks with local LGBTQ + communities. Recruitment involved emailing potential participants a personal invitation to take part in two GMB workshops (the first in December 2021 and the second in February 2022) held via the video-conferencing platform, *Zoom*. A Plain Language Statement and Consent form (PLS-C) was attached to the invitation. The PLS-C outlined the voluntary and confidential nature of participation, details of the GMB workshops, and an outline of withdrawal processes should they wish to no longer be involved in the project. All participants completed, signed, and returned their consent form to the first author prior to attendance at the GMB workshops. A $50 prepaid e-gift card was provided to participants for their time at each workshop.

### The group model building workshops

Two GMB workshops were delivered, which members of the research team facilitated through specific roles: the facilitator (JJL), two notetakers (JH, RA), a modeller (JR), and a psychosocial support person (SE). The facilitator led the workshop activities and discussions, the notetakers provided non-identifiable notes of those discussions, and the modeller coordinated development of the systems map. The systems mapping tool used to support workshop facilitation was STICK-E (Systems Thinking in Community Knowledge Exchange; see: https://sticke.deakin.edu.au/), an online platform developed at Deakin University to assist facilitating GMB with communities. It supports visual mapping with prompts on the definitions of the components of a systems map, along with various formatting tools that are tailored to support delivery of GMB using the set of standardised GMB protocols employed in this workshop design [37–39]. The psychosocial support person provided participants with signposting and linkage to local mental health and wellbeing supports if they felt any distress from their participation during the workshops.

#### GMB Workshop 1 (December 2021)

The first GMB workshop consisted of two activities: (1) Graphs Over Time, and (2) Connection Circles (see: 35). In the Graphs Over Time activity, participants identified what they viewed as important factors influencing regional LGBTQ + people’s access to quality PHC in the local community. Participants then drew individual graphs (or similar visual diagrams) for each factor showing how its influence had changed over the last five years to the present (2017–2021/2022), as well as their associated hope and fears for the future. These Graphs Over Time were used to stimulate group discussion about the identified factors, which were visually represented and shared with participants in real-time.

In the next activity, Connection Circles, participants pointed out how the identified factors were connected in terms of their direction and polarity (i.e., positive or negative). As connections were identified, the modeller (JR) drew these via the online STICK-E tool in real-time. The STICK-E tool then converted the connected factors into a systems map that visually represented the group’s shared understanding of the interconnected factors influencing LGBTQ + people’s access to quality PHC in the region over the last five years.

#### Preparation for GMB Workshop 2 (January 2022)

In preparation for the second GMB Workshop, the research team reviewed the notes from the first GMB Workshop and updated the systems map to ensure that participants’ views were fully captured. Colour coding was used to distinguish between those parts of the systems map that were created during the first GMB Workshop and those parts updated by the research team, based on the workshop notes, in preparation for the second workshop.

#### GMB Workshop 2 (February 2022)

The second and final GMB workshop consisted of initially presenting the updated systems map to the participants, highlighting where the updates were made after reviewing the notes from GMB Workshop 1. All but one participant from GMB Workshop 1 took part in GMB Workshop 2. One participant in GMB Workshop 2 did not participate in GMB Workshop 1. Participants were invited to review, discuss, and update the systems map to ensure its completeness and accuracy. On this final systems map, participants indicated (using a tick – “✓”) where past actions have focused on, as well as where they see future action (using up to three hearts – “❤”) is needed. The concentration of hearts on the final systems map indicated prioritisation of these future action areas, which were then discussed amongst participants.

#### Post-GMB Workshop 2 Review (February 2022)

The research team reviewed the notes from the second GMB workshop to ensure completeness and accuracy of the information captured in the final systems map. A list of potential action areas and priorities identified from the workshop were also compiled from the notes. The research team identified potential key feedback loops by iteratively comparing drafts of the map with the notes to draw out the main stories prioritised by the participants. The final systems map, together with the list of potential action areas and priorities, were provided to the participants for member checking. Participants did not provide any suggested edits.

## Results

### Factors, domains, and interconnections

**Fig. 1:**
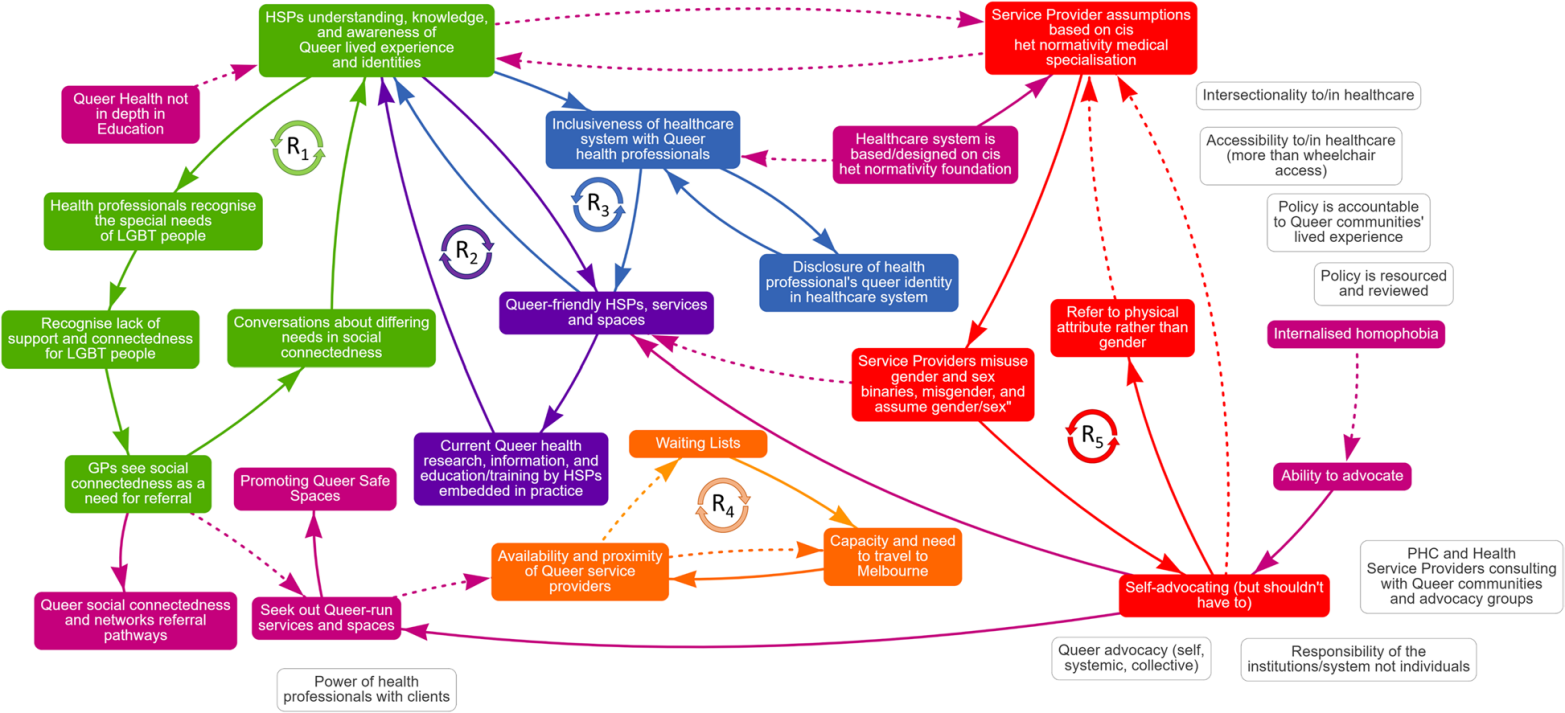
Map of “Regional LGBTQ + primary healthcare support system” from the Participants’ Perspectives**.***Note*. PHC = Primary Healthcare; HSP = Health Service Providers; LGBT = Lesbian, Gay, Bisexual, Transgender. Solid arrows indicate connected factors that change in the same direction (i.e., higher/higher and lower/lower). Dotted arrows indicate connected factors that change in opposite directions (i.e., higher/lower and lower/higher). R1 = Feedback Loop 1: *Aware, Recognise, Talk, Refer, Network* (green arrows and rectangles); R2 = Feedback Loop 2: Power, Disclosure, Inclusiveness (blue arrows and rectangles); R3 = Feedback Loop 3: Current Queer Lived Experience Embedded in Queer Health Services, Research, and Education (purple arrows and rectangles); R4 = Feedback Loop 4: Availability and Capacity of Queer Health Service Providers (orange arrows and rectangles); R5 = Feedback Loop 5: Queer Advocacy Promoting Queer Life and Identities (red arrows and rectangles). All feedback loops are “reinforcing” (“R”). Pink arrows and rectangles represent solely antecedent and/or consequent factors. Black and white rectangles are factors raised but not yet linked with other factors by the participant group

Through the graphs over time and connection circles activities, participants identified 31 factors influencing regional LGBTQ + people’s access to quality primary healthcare in their local community. Two members of the research team (JJL, JR) thematically grouped these factors under key “domains”. These domains were reflexively discussed amongst the rest of the research team in preparation for GMB Workshop 2, and then with the participants during GMB Workshop 2. Changes were made to the key domain groupings where consensus was reached [35]. This process resulted in identifying five key domains, labelled: (1) Queer Literacy, (2) Queer Advocacy, (3) Queer Space, (4) Queer Presence, and (5) Intersectional Queer Life. These five feedback loops can be seen as hypotheses generated collaboratively between the research team and the participants and form the basis for future work to better understand the implications of their behaviour in the system. Brief descriptions of each theme are provided in Table 1. The system map showing the interconnected factors and system-level feedback loops is outlined in Fig. 1.

**Table 1:** Domains, factors, and feedback loops influencing regional LGBTQ + people’s access to quality primary healthcare

Domain	Factor	Feedback Loop/ Antecedent/ Consequence	Action Area Endorsement	
			**Past “**✓**”**	**Future “**❤**”**
Queer Literacy	Queer health not in depth in education	A		❤❤❤
	HSPs understanding, knowledge, and awareness of Queer lived experience and identities	R1, R2, R3, A		
	Health professionals recognise the special needs of LGBT people	R1	✓✓✓✓	
	Recognise lack of support and connectedness for LGBT people	R1		❤
	Service provider assumptions based on cis-heteronormativity medical specialisation	R5, A	✓	
	Service providers misuse gender and sex binaries, misgender, and assume gender/sex	R5, A	✓✓✓	
	Refer to physical attribute rather than gender	R5		
	Conversations about differing needs in social connectedness	R1	✓✓✓	❤
	GPs see social connectedness as a need for referral	R1		❤
	Queer social connectedness and networks referral pathways	C		
Queer Advocacy	PHC and HSPs consulting with Queer communities and advocacy groups	n/a	✓	
	Responsibility of the institutions/system not individuals	n/a		
	Queer advocacy (self, systemic, collective)	n/a	✓✓✓✓	❤
	Self-advocating (but shouldn’t have to)	R5, A		
	Ability to advocate	A		
	Internalised homophobia (Queerphobia)	A		
Queer Space	Current Queer health research, information, education/training by HSPs embedded in practice	R2		❤❤❤
	Power of health professionals with clients	n/a		
	Promoting Queer safe spaces	C	✓✓✓	❤
	Queer-friendly HSPs, services, and spaces	R2, R3		❤
	Seek out Queer-run services and spaces	A, C	✓✓	
	Availability and proximity of Queer service providers	R4	✓✓	❤❤
	Waiting lists	R4		
	Capacity and need to travel to Melbourne	R4	✓✓	
Queer Presence	Healthcare system is based/designed on cis-heteronormative foundation	A	✓	❤
	Inclusiveness of healthcare system with Queer health professionals	R3		❤
	Disclosure of health professional’s Queer identity in the healthcare system	R3	✓✓✓	❤❤
Intersectional Queer Life	Intersectionality to/in healthcare	n/a	✓	
	Accessibility to/in healthcare (more than wheelchair access)	n/a	✓✓	
	Policy is accountable to Queer communities’ lived experience	n/a		
	Policy is resourced and reviewed	n/a		

*PHC*  Primary Healthcare, *HSP*  Health Service Providers, *LGBT*  Lesbian, Gay, Bisexual, Trans, Queer = Umbrella term referring to LGBT people of any gender, as well as people of any other minority sexuality and gender identity (e.g., third gender, non-binary, two-spirit); R1 = Feedback Loop 1: *Aware, Recognise, Talk, Refer, Network*; R2 = Feedback Loop 2: Power, Disclosure, Inclusiveness; R3 = Feedback Loop 3: Current Queer Lived Experience Embedded in Queer Health Services, Research, and Education; R4 = Feedback Loop 4: Availability and Capacity of Queer Health Service Providers; R5 = Feedback Loop 5: Queer Advocacy Promoting Queer Life and Identities. All feedback loops are “reinforcing” (R). A = antecedent factor; C = consequent factor. n/a = factors raised by the participant group but not yet linked with other factors. More ticks (“✓”) = greater the focus of past actions; More hearts (“❤”) = greater the need for future action

### Key feedback loops

In reviewing the interconnected factors in the system map, five main feedback loops were identified and labelled: (1) Aware, Recognise, Talk, Refer, Network (green arrows and rectangles); (2) Current Queer Lived Experience Embedded in Queer Health Services, Research, and Education (purple arrows and rectangles); (3) Power, Disclosure, Inclusiveness (blue arrows and rectangles); (4) Availability and Capacity of Queer Health Service Providers (orange arrows and rectangles); and (5) Queer Advocacy Promoting Queer Life and Identities (red arrows and rectangles). These feedback loops appeared to act as core system-level drivers influencing regional LGBTQ + people’s access to quality PHC in the local community. A description of each feedback loop, and their proposed relationship to the LGBTQ + people’s access to quality PHC over the last five years is provided in the following sections.

#### Feedback Loop 1: aware, recognise, talk, refer, network

This feedback loop operated through factors within the Queer Literacy Domain. This feedback loop told the Queerstory of how primary healthcare professionals’ (PHPs’) understanding, knowledge, and awareness of Queer people’s lived experiences and identities positively contribute to greater recognition of Queer people’s special needs, which in turn increased PHP’s recognition of the lack of support and social connectedness that Queer people face in the local community. Through this increased recognition, PHP’s are more likely to see social connectedness as a need for referral to local community supports and lead to more conversations between PHP’s and Queer people about differing needs relating to cultivating greater social connectedness in the community. Finally, through PHP’s having more conversations in this area, their understanding, knowledge, and awareness of Queer people’s lived experiences and identities also increase; thus, closing the feedback loop.

A consequential effect from this feedback loop related to an increase in the networks and referral pathways available to Queer people looking for support from their PHP relating to social connectedness resulting from PHP’s seeing social connectedness as a need for referral. Where PHPs do not see such a need, Queer people seek out Queer-run service spaces which corresponds to greater word-of-mouth promotion of Queer safe spaces within Queer communities.

#### Feedback Loop 2: embed current queer lived experience in healthcare practice

This feedback loop operated through the intersection of the Queer Literacy and Queer Space Domains. This feedback loop told the Queerstory of how greater understanding, knowledge, and awareness of Queer lived experiences and identities (Queer Literacy) can lead to greater Queer-friendly PHPs and services spaces (Queer Space), but all of which is dependent on having the current Queer health research, information, and education/training by PHPs translate or become embedded into their practice (Queer Space). This embeddedness then leading to greater understanding, knowledge, and awareness of Queer lived experiences and identities by PHPs (Queer Literacy).

An antecedent influence on this feedback loop related to the lack of in-depth Queer health content in PHP’s qualifying educational pathways (e.g., bachelor and master degree study), which if increased could help contribute to PHPs understanding, knowledge, and awareness of Queer lived experience and identities (Queer Literacy). Another antecedent influence related to the cis-heteronormative foundation on which the healthcare system is based and designed that reduces the inclusiveness of that system for QHPs (Queer Presence) and the ability of Queer people to access quality PHC in their local communities.

#### Feedback Loop 3: power, disclosure, and inclusiveness in healthcare systems

This feedback loop operated through the intersection of the Queer Space, Queer Presence, and Queer Literacy Domains. This feedback loop told the Queerstory of how having increased numbers of Queer-friendly PHC services spaces (Queer Space) are dependent on the inclusiveness of healthcare systems (Queer Presence). This inclusiveness is reciprocally dependent the power dynamics between healthcare systems and Queer Health Professionals (QHPs) which dictates whether QHPs feel safe in disclosing their Queer identities to their colleagues and the broader community (Queer Presence). While greater presence of Queer-friendly and Queer-run service spaces is dependent on inclusive healthcare systems and Queer-affirming power dynamics which in turn leads to a greater awareness, knowledge, and awareness of Queer lived experience and identities (Queer Literacy). This in turn can foster greater inclusiveness and likelihood of safe disclosure from QHPs (Queer Presence), and ultimately result in more Queer-friendly PHPs and service spaces (back to Queer Space to close the feedback loop).

An antecedent factor to this feedback loop was how healthcare systems are based or designed on cis-heteronormative ideas of sex, sexuality, and gender. Acting specifically on healthcare systems’ inclusiveness supporting QHPs feeling (un)safe to disclose their identities (Queer Presence), the consequential effect related to reinforcing PHC service providers’ cis-heteronormative assumptions and reducing their level of Queer Literacy. Ultimately posing challenge to Queer people’s access to quality PHC in their local communities and consequently needing to seek support in major, urban, Queer-affirming city centres where such services are more likely available.

#### Feedback Loop 4: availability and capacity of queer-run health service providers

This feedback loop corresponded with small sub-set of factors within the Queer Space Domain. This feedback loop told the Queerstory of how, in seeking out Queer-run PHC services, Queer people found that the availability of such services proximal to their local community region was lacking as the number of Queer-run services were minimal. As a result, the waiting lists of local Queer-run services increased which led to Queer people having to consider whether they travel away from their community (at times up to 60 or 70 kms of travel one-way) to access such services in larger metropolitan centres. In making this consideration, Queer people may not have the capacity to take on a large travel commitment in the context of their life demands (e.g., caring responsibilities, travel costs, employment demands) and therefore find themselves whether they access the healthcare they need now or delay into the future.

#### Feedback Loop 5: queer advocacy promoting queer life and identities

This feedback loop operated through the intersection of the Queer Advocacy and Queer Literacy Domains. This feedback loop told the Queerstory of how Queer people often have to self-advocate to access primary healthcare services and receive quality support once access is achieved (Queer Advocacy). A clear sentiment surrounding the ongoing need to self-advocate is that the individual Queer person should not have to do so. This self-advocacy is targeted directly towards challenging PHP’s assumptions built-upon a cis-heteronormative medical frame (Queer Literacy), with this frame leading to greater misuse of gender and sex binary language, misgendering, and biased assumptions around gender and sex (Queer Literacy). The greater the misuse, misgendering, and biased gender/sex assumptions, the greater the burden for Queer people to challenge PHP’s through self-advocacy to promote Queer life and identities (back to Queer Advocacy to close the feedback loop).

Antecedent influences on this feedback loop relate to individual Queer people’s felt ability or confidence to self-advocate given the experienced unequal power dynamic between PHPs and Queer service users, which in part is related to internalised Queerphobia. Consequently, Queer people seek out Queer-run PHC services outside of their local communities (often in major, metropolitan city centres) in order to access quality PHC.

### Action areas

#### Past action areas endorsement

Queer Literacy was the domain that participants endorsed most frequently in terms of past actions in the region over the last five years (*n* = 11 endorsements). Focus areas within this domain included action on “health professionals recognising the special needs of LGBT [lesbian, gay, bisexual, transgender] people”, including having “conversations about differing needs in social connectedness”; as well as on “service provider assumptions based on cis-heteronormativity medical specialisation”, and “service providers misusing gender and sex binaries, misgendering, and assuming gender/sex”. Queer Space was the second most frequently endorsed domain for past actions (*n* = 9 endorsements), acting primarily through “promoting Queer safe spaces”, “seeking out Queer-run services and spaces”, the “availability and proximity of Queer service providers”, and the “capacity and need to travel to [major, metropolitan centres]”.

Queer Advocacy (*n* = 5 endorsements), Queer Presence (*n* = 4 endorsements), and Intersectional Queer Life (*n* = 3 endorsements) were the three least endorsed areas of past action. Actions across these domains focused on “advocacy at the self, systemic, and collective levels”, promoting “[safe] disclosure of health professional’s Queer identity in the healthcare system”, and “accessibility to/in healthcare (more than wheelchair access)” (Table 1).

#### Future action areas endorsement

Participants endorsed four of the five domains (Queer Literacy, Queer Advocacy, Queer Space, and Queer Presence) in which future actions are needed in the region. The most frequently endorsed domain was Queer Space (*n* = 7 endorsements), followed by Queer Literacy (*n* = 6 endorsements), Queer Presence (*n* = 4 endorsements, and Queer Advocacy (*n* = 1 endorsement). Within the Queer Literacy domain, action was needed most to address “Queer health not in depth in [medical/health] education” (*n* = 3 endorsements). In the Queer Space domain, it was ensuring “current Queer health research, information, education/training by Health Service Professionals [is] embedded in practice” (*n* = 3 endorsements) and the “availability and proximity of Queer service providers” (*n* = 2 endorsements). Lastly, in the Queer Presence domain, it was supporting “[safe] disclosure of health professional’s Queer identity in the healthcare system” that needed attention in the region (Table 1).

## Discussion

This article outlines a novel approach to understanding how dynamics created by local systems have influenced regional LGBTQ + people’s access to quality PHC over the last five years and community-identified priorities for action moving forward. It is important to note that the findings discussed here are not intended to represent broader generalisation across all LGBTQ + communities in all regional areas in Australia nor internationally. Rather, the findings point to one local regional community’s views (albeit with a small sample size) on priority issues they are facing in that region of Australia. Participants identified Queer Literacy, Queer Advocacy, Queer Space, Queer Presence, and Intersectional Queer Life all influencing their ability to access quality PHC in their local communities. These “Five Queers” intersected to form a series of system-level feedback loops or Queerstories centred around promoting/resisting Queer Equity within PHC for LGBTQ + people in regional communities. This Queer-led participatory research initiative provided a closer, multi-level view of what an effective and accessible regional PHC support system looks like for LGBTQ + people in regional areas from the perspective of LGBTQ + people themselves.

### Demonstrating the five queers

The findings indicated how increased networks and Queer-friendly referral pathways are reliant on PHC providers’ awareness of the unique needs and lived experiences of LGBTQ + service users (i.e., Queer Literacy and Intersectional Queer Life). This awareness, together with support in accessing the associated referral pathways, may facilitate a greater sense of place and belonging that reduces othering and marginalisation of LGBTQ + communities (i.e., Queer Space, Presence, and Advocacy) [4, 40, 41]. Key for participants in this study, and for PHC providers to consider in their policy and practice, is demonstrating the Five Queers in several different ways with recommendations for PHC providers outlined in Table 2

**Table 2:** Primary Healthcare (PHC) Recommendations for Demonstrating the Five Queers (Queer Literacy, Queer Advocacy, Queer Space, Queer Presence, and Intersectional Queer Life)

Recommendations	
1	Initiate conversation and use language with regional/rural LGBTQ + service users in ways that support them in feeling visible and their identities affirmed (e.g., making conscious attempts to not mis-gender service users)
2	Resist cis-heteronormative assumptions (e.g., assuming a person is cisgendered and/or heterosexual unless told otherwise) and overtly denounce Queerphobia
3	Increase the availability of Queer-friendly and Queer-run PHC services
4	Provide genuine allyship and leadership in building effective and accessible PHC systems for regional LGBTQ + people
5	View LGBTQ + people’s access to quality PHC in regional/rural areas as a basic human right, with a shared responsibility between PHC providers and Queer communities
6	Overtly share responsibility for naming and understanding regional/rural LGBTQ + communities’ healthcare concerns
7	PHC policy development requires active consultation and collaboration with regional/rural LGBTQ + communities and held accountable to their Queerstories (i.e., their intersectional Queer life, identities, and lived experiences)

First, demonstration can start with PHC providers’ Queer Literacy or knowledge about LGBTQ + people’s diverse and intersectional lived experiences in Australia’s regional/rural areas. In practice this looks like the provider initiating conversation and using language with regional LGBTQ + service users in ways that support them in feeling visible and their identities affirmed (i.e., support greater Queer Presence and Intersectional Queer Life). Conscious attempts to not mis-gender service users and avoid acting on cis-heteronormative assumptions is also important (e.g., assuming a person is heterosexual unless told otherwise) to demonstrate Queer Literacy, promote safe Queer Space, and affirm Queer Presence. These findings support previous research regarding PHC workers’ responsibility to self-educate and communicate in ways that promote LGBTQ + inclusion and equity [42–44]. As with the identified future action priority, further work is needed on increasing PHC workers’ Queer Literacy moving forward.

Second, demonstration of the Five Queers might take form in acting on bringing LGBTQ + people in from society’s margins through increasing the availability of Queer-friendly and Queer-run PHC services (i.e., Queer Advocacy to build Queer Space and Presence) which in turn can support deepening PHC providers’ knowledge of Queer life, health, and wellbeing (i.e., deepen their Queer Literacy). The study results indicated that with greater Queer Literacy among PHC providers, the higher the chance for recognition of intersectional Queer identities and lived experiences and the provision of quality PHC support, which in turn creates Queer-friendly spaces and safety for disclosure of individual PHC professionals’ Queer identities, feeding back to increasing PHC providers knowledge of Queer Life and Identities. PHC service spaces are consequently seen as validating and inclusive of diverse groups of people beyond the cis-heteronormative norm as reflected in earlier research [13, 45, 46].

Finally, key to building effective and accessible PHC systems for regional LGBTQ + people is allyship and leadership. While LGBTQ + communities have a long history of advocacy and organisation [47–49], it is necessary that demonstrating the Five Queers is inclusive of PHC providers’ willingness to work with and in response to LGBTQ + lived experiences and intersectional identities. For example, inclusive of PHC providers’ willingness and initiation around resisting gendered and sex-based assumptions, working hard to avoid mis-gendering, and overtly denouncing Queerphobia. LGBTQ + people’s access to quality PHC in regional/rural areas, as a basic human right that should be but is not afforded to all members of the community [50], becomes a shared responsibility between PHC providers and Queer communities.

If PHC providers shoulder the onus of responsibility for creating safe Queer Space and greater Queer Presence via increased Queer Literacy, it may be (as indicated in the current study findings) that LGBTQ + people’s sharing of health care concerns will be more forthcoming. In this way hierarchical power dynamics between PHC providers and the communities they serve may become more equitable with PHC providers more overtly sharing responsibility for naming and understanding regional/rural LGBTQ + communities’ healthcare concerns [5, 51]. While the study findings also indicated toward LGBTQ + communities’ preference for Queer-run PHC services and supports, the reality is that this is resource and funding dependent [44]. PHC policy development requires active consultation and collaboration with regional LGBTQ + communities and held accountable to Intersectional Queer Life and lived experiences [15] (Table 2). Therefore, demonstration of the Five Queers may play an important role in facilitating a greater sense of belonging, social connectedness, and overall state of “Queer Equity” within regional/rural communities.

### Strengths, limitations, and future research directions

This is the first study to the author group’s knowledge that applies a CBSD approach to understanding the factors influencing LGBTQ + people’s access to quality PHC in regional Australia. A key strength of this study lied in the ability to move beyond listing the primary issues, towards exploring their interconnections in forming system-level feedback loops from the perspective of LGBTQ + people themselves. With this knowledge, and another key strength of the study, the Queer-led author group were able to work with LGBTQ + people living in regional/rural areas is developing priority action areas for the future – thus, developing a preliminary agenda for future participatory research.

In considering the study findings it is important to consider the following limits to their interpretation. First, all feedback loops identified were reinforcing in that they promote change within the system. While this is not uncommon in initial participatory system dynamics projects where communities are initially learning the CBSD approach, future research could explore the presence of any balancing feedback loops that resist system change. Future research could also present the feedback loops to a broader audience in order to further differentiate or combine them, leading to clearer structural insights. The GMB workshops could repeated over time to review and update the system map. In this way, regional/rural LGBTQ + communities can iterate on the ideas in this paper over time to strengthen its rigour – both in terms of representativeness of community beliefs and in terms of rigour of system dynamics.

Also adding to the rigour of this study and of system dynamics would be to expand the reach of the participants to more LGBTQ + people in the Geelong-Barwon region, and indeed in other regional/rural areas of Australia and internationally. It is also important to remain mindful that the study participants were predominantly cis-female, White, and of professional class. It will, therefore, add rigour in future research to nuance the findings according to specific sexuality and gender identities and their intersections with other social standpoints (e.g., class, culture, age, geographical location). For example, how might the identified feedback loops and action priorities differ between trans, nonbinary, and gender diverse people in rural-based communities compared with those living in urban cities? Or, what are the experiences of the Five Queers for bisexual, Indigenous Australian women in remote Australia? Such questions require exploration in future research.

## Conclusion

Accessing quality PHC is challenging for many LGBTQ + people in regional/rural areas where geographical and cis-heteronormative/sexist barriers intersect. This study is a first of its kind in applying a Queer-led, participatory CBSD approach to understanding regional/rural LGBTQ + communities’ priority issues in accessing quality PHC and the system-level feedback loops driving challenges to access equity. Importantly, an initial community identified agenda for future action (and research) is also developed related to the intersection of the Five Queers (Queer Literacy, Queer Advocacy, Queer Space, Queer Presence, and Intersectional Queer Life). This initiative has provided a closer, novel, multi-level system view of what an effective and accessible regional PHC support system looks like for LGBTQ + people in non-metropolitan, regional/rural areas from the perspective of LGBTQ + people themselves.

## Data Availability

The datasets during and/or analysed during the current study available from the corresponding author on reasonable request.

## Abbreviations

CBSD: Community-Based System Dynamics
GMB: Group Model Building
GP: General Practitioner
JH: Joshua Hayward
JJL: James J Lucas
JR: Joleen Ryan
LGBTQ +: Lesbian, gay, bisexual, transgender, Queer, and people of any other minority sexuality or gender identity
LGBT: Lesbian, gay, bisexual, transgender
PHC: Primary healthcare
PHP: Primary healthcare professional
PLS-C: PLAIN Language Statement and Consent form
RA: Rojan Hayward
SE: Sarah Epstein
STICK-E: Systems Thinking in Community Knowledge Exchange
WHO: World Health Organization

## Footnotes

1 In this article, the terms LGBTQ + and Queer are used interchangeably with the latter reflecting contemporary reclaiming of the historically derogatory term “Queer” as a resistive and empowering stance against oppressive cis-heteronormative/sexist social norms.
